# Direct and Indirect Costs of Prostate Cancer: A Comprehensive Assessment of Economic and Social Impact

**DOI:** 10.3390/cancers17193257

**Published:** 2025-10-08

**Authors:** Izabela Gąska, Aleksandra Czerw, Monika Pajewska, Olga Partyka, Andrzej Deptała, Anna Badowska-Kozakiewicz, Natalia Czerw, Dominika Mękal, Katarzyna Sygit, Katarzyna Wojtyła-Blicharska, Jarosław Drobnik, Piotr Pobrotyn, Dorota Waśko-Czopnik, Adam Wiatkowski, Michał Marczak, Tomasz Czapla, Ewa Bandurska, Weronika Ciećko, Elżbieta Grochans, Anna M. Cybulska, Daria Schneider-Matyka, Kamila Rachubińska, Remigiusz Kozlowski

**Affiliations:** 1 Medical Institute, Jan Grodek State University in Sanok, 38-500 Sanok, Poland; 2 Department of Health Economics and Insurance, Center for the Humanities and Social Sciences of Medicine, Medical University of Warsaw, 00-581 Warsaw, Poland; 3 Department of Economic and System Analyses, National Institute of Public Health NIH-National Research Institute, 00-791 Warsaw, Poland; 4 Department of Oncology Propaedeutics, Medical University of Warsaw, 01-445 Warsaw, Poland; 5 Students’ Scientific Organization of Cancer Cell Biology, Department of Oncology Propaedeutics, Medical University of Warsaw, 01-445 Warsaw, Poland; 6 Faculty of Medicine and Health Sciences, University of Kalisz, 62-800 Kalisz, Poland; 7 Department of Family Medicine, Faculty of Medicine, Wroclaw Medical University, 51-141 Wroclaw, Poland; 8 Pulsantis Specialist and Rehabilitation Clinic Ltd., 53-238 Wroclaw, Poland; 9 Department of Gastroenterology, Hepatology with Inflammatory Bowel Disease Subunit, Provincial Specialist Hospital J. Gromkowskiego, 51-149 Wroclaw, Poland; 10 Department of Non-Surgical Clinical Sciences, Faculty of Medicine, Wroclaw University of Science and Technology, 50-370 Wroclaw, Poland; 11 Faculty of Medicine, Wroclaw Medical University, 50-345 Wroclaw, Poland; adam.wiatkowski@student.umw.edu.pl; 12 Collegium of Management, WSB University in Warsaw, 03-204 Warsaw, Poland; 13 Department of Management, Faculty of Management, University of Lodz, 90-237 Lodz, Poland; tomasz.czapla@uni.lodz.pl; 14 Center for Competence Development, Integrated Care and e-Health, Medical University of Gdansk, 80-204 Gdansk, Poland; 15 Department of Nursing, Faculty of Health Sciences, Pomeranian Medical University in Szczecin, 71-210 Szczecin, Polandanna.cybulska@pum.edu.pl (A.M.C.);; 16 Department of Management and Logistics in Healthcare, Medical University of Lodz, 90-131 Lodz, Poland; remigiusz.kozlowski@umed.lodz.pl

**Keywords:** prostate cancer, costs, cost-effectiveness, metastatic prostate cancer

## Abstract

**Simple Summary:**

Prostate cancer is the second most common malignancy among men, and the number of new cases is projected to increase substantially in the coming years. To assess the current state of knowledge on the economic burden of this disease, we conducted a literature review that included 31 studies. Our analysis focused on estimates of direct costs—such as treatment, adjuvant and neoadjuvant therapies, and supportive and palliative care—as well as indirect costs. However, the vast majority of studies addressed direct costs only, highlighting a clear gap in the literature. This accentuates the need for further comprehensive reviews and the development of standardized methodologies to enable reliable comparisons across studies.

**Abstract:**

Background: Prostate cancer is the second most common malignant cancer among men, and according to the predictions, the estimated number of new cases will substantially grow in the coming years. Therefore, the costs of the disease will increase as well. Methods: We conducted a literature review of the state of knowledge about the costs of treatment and the economic burden of prostate cancer. The vast majority of studies were focused on direct costs only, which clearly shows the literature gap. Results: We focused on the estimates of direct costs, i.e., treatment of prostate cancer, adjuvant and neoadjuvant treatment, and supportive and palliative care, and indirect costs. Cost-effectiveness analyses indicated that docetaxel combined with androgen deprivation therapy (ADT) was the most cost-effective strategy for metastatic hormone-sensitive prostate cancer (incremental cost-effectiveness ratio (ICER): USD 13,647). In contrast, novel therapies such as PARP inhibitors and whole-genome-sequencing-guided treatments were not cost-effective unless drug prices were reduced by 47–70%. In the United States, 5-year cumulative treatment costs ranged from USD 48,000 for conservative management to over USD 91,000 for radiotherapy, while out-of-pocket expenses averaged AUD 1172 in Australia. Indirect costs were also considerable, with Slovakia reporting an increase in sick leave costs from EUR 1.2 million in 2014 to EUR 2.1 million in 2022. Conclusions: Metastatic hormone-sensitive prostate cancer and metastatic castration-resistant prostate cancer were the most frequent categories for various treatment cost evaluations. A few specific combinations of drugs were cost-effective only under the condition of dropping the unit prices of a medication. Further summarizing, reviewing, and developing a methodology for standardized comparisons are needed.

## 1. Introduction

Prostate cancer is the second most common malignant cancer among men, following lung cancer. Prostate cancer is the fifth leading cause of cancer-related death among men. According to World Health Organization data, in 2022, there were 1,467,854 new cases worldwide [1], corresponding to an age-standardized incidence rate of 29.4 per 100,000. The highest incidence was reported in France, at 157.5 per 100,000. That same year, there were 397,430 deaths globally, with an age-standardized mortality rate of 7.3 per 100,000.

The five-year survival rate is high—around 90%—in countries such as Austria, Germany, Finland, Belgium, France, and Portugal; however, it is considerably lower (60–70%) in Denmark, Latvia, Poland, and Slovakia. The lowest survival rates, between 50% and 60%, are observed in Bulgaria. While in certain European countries the 5-year survival after prostate cancer is 90% or lower, the United States exhibits much higher overall 5-year relative survival, approaching 97–98%. Survival exceeds 99% for localized and regional disease but drops substantially for metastatic cases (to ~37–38%).

According to projections from the International Agency for Research on Cancer [2], the number of new cases is expected to rise substantially in the coming years, particularly in Africa, Latin America, and Asia (see Table 1).

The main risk factors for prostate cancer include age, family history, and genetic predisposition [3]. Also, additional factors like smoking, diet, physical activity, specific medications, and occupational factors may contribute to developing prostate cancer [4,5]. The occupational factors include agricultural workers, petroleum workers, and rotating night shift work [5].

Diagnostics and screening tests include a rectal examination, a prostate-specific antigen (PSA) test, and transrectal ultrasound of the prostate gland, as well as magnetic resonance imaging (MRI) [6]. The treatment methods of prostate cancer include active surveillance, chemotherapy, radical prostatectomy, radiotherapy, brachytherapy, hormone therapy, transurethral resection of the prostate, immunotherapy, proton beam therapy, radiopharmaceutical treatment, high-intensity focused ultrasound, and cryotherapy. Radical prostatectomy and radiation therapy are the main treatment modalities for lower stages of the localized disease. Chemotherapy, hormone therapy, or immunotherapy are mainly used in more advanced stages.

Prostate cancer constitutes a chronic condition necessitating prolonged therapeutic management, which entails substantial financial burden and frequently imposes limitations on patients’ social functioning and occupational activity [3]. From a health economics perspective, both direct and indirect costs should be considered. Direct costs encompass healthcare system expenditures associated with diagnostic procedures, medical interventions, and follow-up care, as well as patients’ out-of-pocket expenses for pharmacotherapy and medical supplies. Indirect costs primarily reflect productivity losses attributable to sickness-related work absence and diminished work performance (presenteeism) [7]. Therefore, direct costs are costs that are directly attributable to patient care, and indirect costs are costs that are not directly related to patient care. This distinction is not country-specific and can be applicable globally. It helps to realize that the costs of any disease are higher than the costs of medical procedures and resources necessary for their application. Prostate cancer is a chronic disease that often requires prolonged and invasive treatment. The physical toll of these treatments frequently limits professional productivity and social engagement, particularly for men in their prime working years. Since, according to the Global Cancer Observatory prognosis, the incidence of prostate cancer will increase, the larger number of patients will lead to higher treatment costs and a growing loss of productivity due to sickness absence. The objective of this article is to present the latest available knowledge on the direct and indirect costs of prostate cancer as a burden for healthcare financing.

Two reviews on the subject were published in 2022. One was focused on metastatic hormone-sensitive and non-metastatic castration-resistant prostate cancer in the Canadian context [8]. The second one was based on articles describing the economic burden and cost-effectiveness of treatment in OECD countries [9]. Also, the second review focused primarily on the earlier stages of the disease and so, in a sense, is complementary to the first review. The first review concluded that docetaxel in combination with androgen deprivation therapy was the most cost-effective treatment for patients with metastatic hormone-sensitive prostate cancer. Apalutamide, darolutamid, and enzalatumide, on the other hand, were cost-effective for non-metastatic castration-resistant prostate cancer. The second review, based on 13 articles, underlined that the direct cost of a treatment was the main driver for treating less severe prostate cancer cases, while the loss in productivity was most important in more severe cases. In our review, we start with the year 2022 to cover the papers that were not included in the previous reviews.

## 2. Materials and Methods

A literature review was conducted to summarize the state of knowledge about the costs of treatment and the economic burden of prostate cancer within MEDLINE, Health Source—Academic Edition, and Academic Search Ultimate databases. We applied the following query: (Neoplasm or cancer or carcinoma) AND (prostate) AND (cost OR costs OR economic analysis OR economic evaluation OR economic loss OR expenditure OR spend OR expense OR burden OR productivity OR costs OR cost analysis). The scope was limited to papers published during the 2022–2025 period. We also applied additional filters limiting the papers acquired to those focused on humans and providing the answers to clinical questions focusing on costs and economics. The criteria for inclusion of publications according to the PICOS scheme are presented in Table 2.

**Figure 1:**
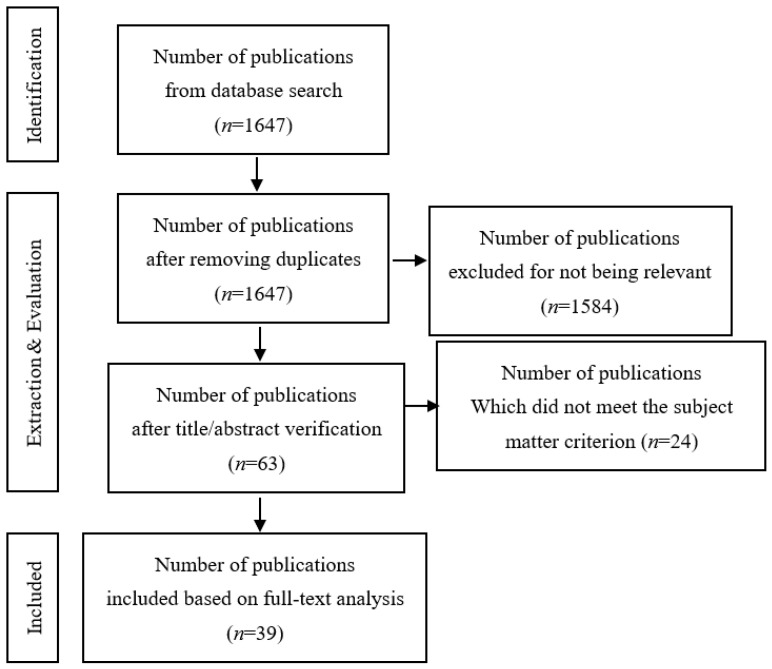
Simplified PRISMA scheme for inclusion of publications for further review.

The search performed within the records of the MEDLINE database led to 1647 papers. After title and abstract verification for being relevant to the subject, we limited the number of articles to 63. After full-text assessment, 39 studies met the inclusion criteria and were included in the final analysis. The study selection process is illustrated in the PRISMA flow diagram in Figure 1.

## 3. Results

Table 3 presents a summary of the publications included in the review with a description of the most important features.

The review of the results acquired in the studies included is divided into sections for direct costs, i.e., treatment of prostate cancer, adjuvant and neoadjuvant treatment, and supportive and palliative care, and indirect costs. The papers resulting from the current review are, above all, focused on the therapy of advanced (metastatic) disease.

### 3.1. Direct Costs

#### 3.1.1. Treatment of Prostate Cancer

Clinical trials have confirmed the effectiveness of combining an androgen receptor signaling inhibitor (ARSI) with docetaxel and androgen deprivation therapy (ADT) in the treatment of metastatic hormone-sensitive prostate cancer. Nevertheless, simulation studies [10] indicated that only the docetaxel–ADT doublet was cost-effective, with an incremental cost-effectiveness ratio of USD 13,647. The high cost of ARSIs remains a limiting factor. The cost of ARSIs, which is expensive, needed to be discounted by 47–70% to improve cost effectiveness in the USA, UK, and Australian health sectors.

When comparing seven treatments in metastatic hormone-sensitive prostate cancer, abiraterone acetate–prednisone androgen deprivation therapy was found to be the most cost-effective strategy in the United States [11]. The estimated 10-year average costs ranged from USD 34,349 for androgen deprivation therapy (ADT) alone to USD 658,928 for darolutamide–docetaxel–ADT. Mean quality-adjusted life years (QALYs) varied from 3.25 for ADT to 4.57 for enzalutamide–ADT.

Polymerase (PARP) inhibitors that provide benefits in terms of survival were not found to be cost-effective as they were associated with additional survival of 0.19 QALYs when compared to the standard care, and at the same time with an additional cost of CAD 565,383/QALY [12].

In metastatic castration-resistant prostate cancer, whole-genome sequencing (WGS)-guided systemic therapy expanded treatment eligibility, identifying an additional 21% of patients as suitable for personalized treatment with PD-1/PD-L1 inhibitors or olaparib, resulting in an incremental cost-effectiveness ratio (ICER) of EUR 289,625 per QALY gained in the Netherlands [13]. WGS becomes cost-effective if the costs of biomarker-guided therapies are reduced by approximately 62%.

In a study conducted in Australia [14], BRCA testing-guided olaparib treatment was found to lead to an incremental cost of AUD 7841 and a gain of 0.06 QALYs. The incremental cost-effectiveness ratio (ICER) was AUD 143,613 per QALY. Also, the likelihood of cost-effectiveness increased to 66 percent if the price of olaparib was reduced by 30 percent.

For the management of advanced prostate cancer, the early use of androgen receptor pathway inhibitors (ARPIs) was found to be most cost-effective for a cost-effectiveness threshold (CET) of CAD 100 K per QALY [15]. For a widely accepted threshold of CAD 50 K per QALY, the early treatment is most cost-effective in metastatic castration-sensitive prostate cancer and not cost-effective for non-metastatic castration-sensitive prostate cancer.

Another study [16] evaluated the use of ADT + docetaxel + ARPIs combinations in the course of treatment of metastatic hormone-sensitive prostate cancer and found their use to be justified only in synchronous, high-volume mHSPC in resource-rich settings. The triplets were found to be too costly in resource-limited settings, particularly when compared to doublets like ADT + ARPIs.

Also, regarding the population of patients with metastatic hormone-sensitive prostate cancer, the cost-effectiveness of rezvilutamide combined with androgen deprivation therapy and the cost-effectiveness of bicalutamide combined with androgen deprivation therapy were assessed [17]. The rezvilutamide group showed an expected gain of 2.28 QALYs and an incremental cost of USD 60,758.82 compared with the bicalutamide group. The ICER for the rezvilutamide group compared to the bicalutamide group was USD 26,656.94 per QALY within the Chinese healthcare system.

In another study [18], the cost-effectiveness of triptorelin, goserelin, and leuprolide in the treatment of patients with metastatic prostate cancer in Iran was compared. The mean costs for goserelin, triptorelin, and leuprolide treatments were USD 13,539.13 and 6.365 quality-adjusted life-years (QALYs), USD 18,124.75 and 6.658 QALYs, and USD 26,006.92 and 6.856 QALYs, respectively. Therefore, goserelin was considered to be the best treatment option on the basis of the incremental cost-effectiveness ratio.

Enzalutamide was found to be associated with a high incremental cost-effectiveness ratio (ICER) of USD 6260 per QALY gained when compared to abiraterone in Iran [19]. The budget impact of enzalutamide for the Iranian health system for 5 years was equal to USD 6,362,127.

Table 4 depicts the summary of the results for treatments expressed as currency/QALY.

A study conducted in England evaluated extending gonadotropin-releasing hormone agonist (GnRHa) therapy administration from every month or 3 months to 6 months [20]. Modifying the mode of administration resulted in estimated annual cost savings of GBP 5,164,296, achieved through a reduction of 148,478 appointments and 37,119 appointment-hours.

Analysis of a cohort of 74,324 patients in the United States revealed that cumulative total and out-of-pocket costs were substantially higher for radiation therapy and surgical treatment compared with conservative management [21]. This finding was consistent according to calculations for 1, 3, and 5 years after diagnosis. The total cumulative cost estimates for conservative management in years 1/3/5 were estimated to be equal to USD 15,896/USD 33,436/USD 48,110, while the cumulative patient out-of-pocket costs were equal to USD 2003/USD 4540/USD 6621. The cumulative total costs for surgery in years 1/3/5 were equal to USD 38,348/USD 49,424/USD 60,885, and the out-of-pocket costs were equal to USD 2980/USD 5255/USD 7221. The cumulative total costs for radiation in years 1/3/5 were equal to USD 65,397/USD 77,859/USD 91,497, and the cumulative out-of-pocket costs were equal to USD 3151/USD 5481/USD 7504, respectively. Another study estimating the costs of treatment for localized non-metastatic prostate cancer depending on the treatment modality [22] in the United States showed that proton-beam therapy was associated with the highest total cost, while brachytherapy was associated with the lowest cost. At the same time, the use of brachytherapy decreased between 2009 and 2021, and the use of proton-beam therapy was rare (0.1%).

In another study cost-effectiveness results of magnetic-resonance-guided transurethral ultrasound ablation, robot-assisted radical prostatectomy, external beam radiation therapy, and active surveillance were compared [23]. Active surveillance, i.e., a monitoring approach with regular tests to check for changes without immediate treatment like surgery or radiation, was associated with the highest number of QALYs (12.67), followed by magnetic-resonance-guided transurethral ultrasound ablation (12.35), external beam radiation therapy (12.35), and then robot-assisted radical prostatectomy (12.20). On the other hand, robot-assisted radical prostatectomy was associated with the lowest costs (EUR 46,997) over one patient’s lifetime, while magnetic-resonance-guided transurethral ultrasound ablation was a more expensive alternative (EUR 48,826). The incremental cost-effectiveness ratio of active surveillance compared with robot-assisted radical prostatectomy was EUR 11,600 per QALY, and that of magnetic resonance-guided transurethral ultrasound ablation compared with robot-assisted radical prostatectomy was equal to EUR 12,193 per QALY.

Active surveillance was also proven to be more cost-effective than radical prostatectomy and radiation therapy for patients with a life expectancy shorter than 10 years [24]. However, it was not true in the case of patients with longer life expectancy.

Low fraction with ultra-hypofractionated radiation therapy in the treatment of intermediate-to-high-risk prostate cancer was not found to be more cost-effective than the conventional fraction treatment in a six-year perspective [25].

Focal therapy, which treats specific areas of tumor in non-metastatic prostate cancer in patients unsuitable for active surveillance, led to a lower overall cost and higher QALY gains than prostatectomy or external beam radiotherapy [26]. Incremental net monetary benefit (INMB) values confirmed focal therapy as cost-effective versus the alternative treatment.

External beam radiotherapy was found to be the most cost-efficient treatment for low-risk prostate cancer, with costs of EUR 2492 per treatment [27]. For intermediate-risk prostate cancer, differences between moderate hypofractionation and brachytherapy were found to be small and fall within the range of EUR 4638–5140. For high-risk prostate cancer, differences between radical prostatectomy and radiotherapy with androgen deprivation therapy fall within the range EUR 7087–7474.

In a systematic review [28], intensity-modulated radiation therapy was found to be more cost-effective than three-dimensional conformal radiation therapy in terms of incremental cost-effectiveness ratio. Radiotherapy was found to be more effective than radical prostatectomy.

In a study evaluating robotic platforms [29] for robot-assisted radical prostatectomy, the da Vinci system was associated with higher total costs than the Hugo system. The costs were equal to EUR 497.21 and EUR 3511.73, respectively. However, excluding the cost of surgery, the da Vinci platform was less expensive in operation. The costs were equal to EUR 1481.18 and EUR 1926.18, respectively.

In another study [30], the economic burden associated with disease progression from metastatic castration-sensitive to castration-resistant prostate cancer on treatment in the United States was estimated. When progressing, mean all-cause total healthcare costs per patient per month increased from USD 4424 (medical costs: USD 2846) to USD 9717 (medical costs: USD 4654), and mean cancer-related total healthcare costs per patient per month increased from USD 2859 (medical costs: USD 1626) to USD 8012 (medical costs: USD 3285).

Total medical costs of treating prostate cancer in Switzerland were estimated at CHF 347 million [323–372] in 2018 [31]. They were equal to 0.45% of total healthcare spending in Switzerland in 2018. Treatment of metastatic prostate cancer took two-thirds of the spending.

In the United States the mean total all-cause and PC-related costs after starting first-line therapy were estimated to be equal to USD 111,060 and USD 99,540 per patient per year, respectively. After starting the treatment, a patient had 1.2 inpatient admissions, 1.1 emergency room visits, and 27.6 outpatient visits per year on average [32].

Also, in the United States the costs of treatment for patients diagnosed with metastatic castration-resistant prostate cancer after starting first-line therapy were estimated to be equal to USD 13,746 for all-cause costs and USD 12,061 for prostate-cancer-related costs per patient per month [33]. The first-line therapy, on average, lasted for 8.5 months. For patients diagnosed with metastatic castration-sensitive prostate cancer the estimated costs of treatment in the United States were equal to USD 2551 for all-cause costs per patient per month and to USD 839 for PC-related costs per patient per month [34], and in 15 months follow-up mean all-cause costs were equal to USD 5950 per patient per month and PC-related costs were equal to USD 4363 per patient per month.

The costs associated with treatment of non-metastatic castration-resistant prostate cancer were evaluated in Italy [35]. They were equal to EUR 4710 per patient per year on average. The estimated range for the cost was quite large and it fell between EUR 2243 and EUR 8423. The impact of diagnostic imaging on the cost was highest.

The costs of treatment in a longitudinal perspective were evaluated on a large sample of 29,614 patients diagnosed with prostate cancer [36]. Prostate cancer costs were highest in the first year after diagnosis and the year before death. They were equal to USD 14,307.9 (95% CI: USD 13,970.0, USD 14,645.8) and USD 9959.7 (USD 8738.8, USD 1181.0), respectively. For advanced-stage PCa, primary treatment with radiation therapy involved significantly higher costs than a radical prostatectomy or other surgeries. Androgen deprivation therapy had the highest cost for high-grade and early-stage cancer during the first three years after diagnosis.

In a study conducted in Australia [37], out-of-pocket costs associated with prostate cancer within 6 months of diagnosis were estimated. They were equal to AUD 1172 (AUD 343–2458). Higher Gleason score and multiple comorbidities involved higher costs.

In a study conducted in Spain [38], the direct costs associated with prostate cancer were evaluated on the basis of medical records from the period between 2016 and 2020. The mean annual cost of a single admission was estimated to be equal to EUR 5212.98, and prostate resection was the most frequent medical procedure applied.

#### 3.1.2. Adjuvant and Neoadjuvant Treatment

Additional radiotherapy was associated with 0.92 incremental QALYs with increased costs of USD 26,098 with an incremental cost-effectiveness ratio (ICER) of USD 28,452/QALY when compared to the standard of care for patients with only non-regional lymph node metastases and 3.83 incremental QALYs with increased costs of USD 153,490 with an ICER of USD 40,032/QALY for patients with up to three bone metastases [39].

The costs of adjuvant and neoadjuvant treatment were also compared to each other [40]. The cost of adjuvant chemotherapy was USD 4174 higher than the cost of neoadjuvant chemotherapy. The 5-year biochemical recurrence-free rates for adjuvant chemotherapy and neoadjuvant chemotherapy were equal to 59% and 86%.

The cost-effectiveness of exercise interventions as adjuvant therapy was examined as well [41]. High- and low-to-moderate-intensity physical exercises were evaluated. No significant differences regarding the intensity of exercise were detected. High-intensity exercises involved a cost of EUR 27,314 in the perspective over 12 months, while low-to-moderate-intensity exercises had a cost of EUR 29,788. Regarding QALYs for high- and low-to-moderate-intensity exercises, they were equal to 1.190 and 1.185, respectively.

#### 3.1.3. Supportive and Palliative Care

In a study conducted in Australia [42], a health-service-based program and a telenursing service for men living with the burden of prostate cancer for many years were evaluated in terms of social return on investment. The Prostate Cancer Specialist Nursing Programs resulted in an SROI ratio of 1:1.62 for the health-service-based program. This means that, for every dollar invested, a return of AUD 1.62 was obtained. For the telenursing program, the SROI ratio was equal to 1:2.34, which means that, for every dollar invested, a return of AUD 2.34 was obtained.

### 3.2. Indirect Costs

In a retrospective study conducted in Slovakia [43], both direct and indirect costs were estimated. The incidence of prostate cancer as a percentage of all cancer diagnoses was found to rise from 5.94% in 2012 to 7.66% in 2021. The rates of mortality in 2012 and 2021 were similar and equal to 5.31% and 5.29%, respectively. The direct costs increased from EUR 20,969,865 in 2014 to EUR 54,105,198 in 2022. The indirect costs involved sick leave and disability. The costs of sick leave increased from EUR 1,199,672 in 2014 to EUR 2,122,042 in 2022. The costs of disability increased from EUR 1,789,115 in 2014 to EUR 3,019,661 in 2022. This gives a total cost increase equal to EUR 1,230,546.

In a study focused on estimating financial burden in the Iranian population, the cost from the perspective of society was estimated to be equal to USD 54 million. Also, 5.44% of patients were found to be seriously impoverished due to the cost associated with prostate cancer [44]. In another study [45], indirect costs from productivity losses were estimated to be equal to USD 209,760 for a patient.

The cost in days off work due to prostate cancer was evaluated based on a sample of 6693 patients [46]. It was found that surgical patients were more likely to report more than 7 days off work in the first year; however, this proportion decreased later. Patients undergoing radiation were less likely to need time off from work.

In a study based on a survey with 495 patients, the indirect costs ranged from USD 154.00 to USD 717.40 for a month and were higher for disease with a higher risk [47].

The authors of a qualitative study based on interviews with Black prostate cancer survivors and their caregivers [48] regarding indirect costs concluded that the weight of indirect costs varied and they also found indirect costs to be interconnected, which depicts the serious impact of financial well-being on prostate cancer survivors and caregivers.

## 4. Limitations

The current paper is based on a systematic review methodology. Definitely, the conclusions regarding indirect costs are limited because of the limited accessibility of papers focusing on the matter. Perhaps a specific publication bias exists. Indirect costs differ between countries as healthcare systems do. Each evaluation of indirect costs is country-specific and therefore it is more difficult for authors to reach a larger audience of readers from other countries. Also, the papers included in our review are the papers included in three databases only, and their content may overlap. Furthermore, the evaluations of direct costs from different studies, if expressed as a currency, use different units, which makes comparison difficult as the relative value of each currency constantly changes over time.

The vast majority of studies focused on the direct costs of treatment. Only three of the thirty-nine publications addressed adjuvant and neoadjuvant therapies, and just six examined indirect costs, highlighting a clear literature gap in evaluating the indirect economic burden of prostate cancer—an area that remains largely overlooked. Most analyses of direct costs were conducted for specific diagnostic categories, with metastatic hormone-sensitive prostate cancer and metastatic castration-resistant prostate cancer being the most frequently assessed. Several studies indicated that certain drug combinations are cost-effective only if unit prices are significantly reduced. Comparisons of treatment cost-effectiveness remain challenging due to methodological complexity: studies employ different cost units, vary across countries, and are influenced by diagnostic categories and disease stage. Furthermore, the papers cover the costs in a limited way. They focus on a specific treatment only, which does not give a comprehensive view of their impact on PCa costs, and they did not take indirect costs into account. Also, the comparison of indirect costs would be difficult since no widely accepted definitions and methods to assess presenteeism have been developed; however, guidelines were published [49]. The same is true for absenteeism, for which the cost can be estimated with either the approach based on human capital or friction costs [50]. The methodology for estimating indirect costs for various diseases was published [51,52,53].

## 5. Conclusions

The estimated number of new cases of prostate cancer will substantially grow in the coming years, which inevitably will lead to an increase in all types of costs. Greater attention should be directed toward the societal burden of prostate cancer, including loss of productivity, the caregiving demands placed on family members, and the psychological distress associated with both the disease and its treatment. Therefore, summarizing, reviewing, and developing a methodology for standardized comparisons are necessary.

## Figures and Tables

**Table 1:** Estimated number of new cases of prostate cancer from 2022 to 2045 in males aged 0–85+.

	Africa		Latin America and the Caribbean		Northern America		Europe		Oceania		Asia	
	n	Risk (%)	n	Risk (%)	n	Risk (%)	n	Risk (%)	n	Risk (%)	n	Risk (%)
2022	103,050	0	225,985	0	255,782	0	473,011	0	23,602	0	386,424	0
2025	110,643	7.4	239,232	5.9	272,553	6.6	489,130	3.4	25,703	8.9	414,466	7.3
2030	133,920	30.0	280,641	24.2	293,158	14.6	530,572	12.2	28,564	21.0	499,271	29.2
2035	162,125	57.3	325,568	44.1	307,888	20.4	564,578	19.4	31,259	32.4	591,547	53.1
2040	196,021	90.2	372,984	65.0	319,630	25.0	592,417	25.2	33,927	43.7	686,816	77.7
2045	236,039	129.1	421,935	86.7	331,610	29.6	613,774	29.8	36,688	55.4	781,126	102.1
2050	282,005	173.7	471,395	108.6	345,571	35.1	628,400	32.9	39,616	67.9	870,939	125.4

Source. Global Cancer Observatory; n—number of new cases; Risk (%)—risk percentage, i.e., number of new cases in the year of analysis as a percentage of 2022 case base.

**Table 2:** Criteria for inclusion of publications according to the PICOS scheme.

Population (P)	Patients Diagnosed with Prostate Cancer
Intervention (I)	Costs, economics
Comparator (C)	Any or none
Outcomes (O)	Direct costs of prostate cancer treatment, indirect costs of prostate cancer, economic burden
Studies (S)	Case studies, prospective studies, retrospective studies, systematic reviews, RCTs
Limitations	Publications in English assessing the impact of prostate cancer on the quality of life, publication period 1 January 2022–30 May 2025
Exclusion	Non-English publications, studies not directly linked to prostate cancer

**Table 3:** Characteristics of publications included in the review.

Author/Year	Country	Currency	Measure	Methodology	Type of Costs	Group of Patients
Sathianathen, N. et al., 2024 [10]	USA, UK Australia	USD	ICER	Cost-effectiveness analysis	Direct costs	Patients with metastatic hormone-sensitive prostate cancer
Yoo, M. et al., 2023 [11]	USA	USD	ICER, QALY	Cost-effectiveness analysis	Direct costs	Patients with metastatic hormone-sensitive prostate cancer
Yanev, I. et al., 2025 [12]	Canada	CAD	ICER, QALY	Cost-effectiveness analysis	Direct costs	Patients with metastatic prostate cancer resistant to castration
Fu, J. et al., 2025 [13]	The Netherlands	EUR	ICER, QALY	Cost-effectiveness analysis	Direct costs	Patients with castration-resistant prostate cancer
Teppala, S. et al., 2024 [14]	Australia	AUD	ICER, QALY	Cost-effectiveness analysis	Direct costs	Patients with metastatic prostate cancer resistant to castration
Litvin, V. et al., 2025 [15]	Canada	CAD	ICER, QALY	Cost-effectiveness analysis	Direct costs	Patients with castration-sensitive prostate cancer, metastatic and non-metastatic
Ávila, C. et al., 2025 [16]	Chile	USD	ICER	Cost-effectiveness analysis	Direct costs	Patients with metastatic hormone-sensitive prostate cancer
Ding, H. et al., 2024 [17]	China	USD	ICER, QALY	Cost-effectiveness analysis	Direct costs	Patients with metastatic hormone-sensitive prostate cancer
Rezaee, M. et al., 2024 [18]	Iran	USD	QALY	Cost-effectiveness analysis	Direct costs	Patients with metastatic prostate cancer
Goudarzi, Z. et al., 2024 [19]	Iran	USD	ICER	Cost-effectiveness analysis	Direct costs	Patients with metastatic prostate cancer resistant to castration
Cornford, P. et al., 2023 [20]	UK	GBP	-	Cost-effectiveness analysis	Direct costs	Patients with prostate cancer
Housten, A. et al., 2025 [21]	USA	USD	-	Assessment of the cost of the disease	Direct costs	Patients with prostate cancer
Sebastian, N. et al., 2025 [22]	USA	USD	-	Assessment of the cost of the disease	Direct costs	Patients with localized non-metastatic prostate cancer
Muhler, P. et al., 2025 [23]	UK	EUR	ICER, QALY	Cost-effectiveness analysis	Direct costs	Patients with localized prostate cancer
Naser-Tavakolian, A. et al., 2023 [24]	USA	USD	QALY	Cost-effectiveness analysis	Direct costs	Patients with clinically localized prostate cancer
Sun, S. et al., 2023 [25]	Sweden	SEK	ICER, QALY	Cost-effectiveness analysis	Direct costs	Patients with prostate cancer
Reddy, D. et al., 2023 [26]	UK	GBP	QALY, INMB	Cost-effectiveness analysis	Direct costs	Patients with non-metastatic prostate cancer unsuitable for active surveillance
Moll, M. et al., 2023 [27]	Switzerland	EUR	-	Assessment of the cost of the disease	Direct costs	Patients with prostate cancer
Adel, A. et al., 2024 [28]	USA	USD	ICER	Systematic review	Direct costs	Patients with prostate cancer
Landi, S. et al., 2025 [29]	UK	EUR	-	Assessment of the cost of the disease	Direct costs	Patients with organ-confined prostate cancer
Kaye, D. et al., 2024 [30]	USA	USD	-	Cost-effectiveness analysis	Direct costs	Patients with metastatic prostate cancer
Stucki, M. et al., 2024 [31]	Switzerland	CHF	-	Assessment of the cost of the disease	Direct costs	Patients with prostate cancer
Swami, U. et al., 2023 [32]	USA	USD	-	Assessment of the cost of the disease	Direct costs	Patients with prostate cancer
Kaye, D. et al., 2024 [33]	USA	USD	-	Assessment of the cost of the disease	Direct costs	Patients with metastatic castration-resistant prostate cancer
Kaye, D. et al., 2024 [34]	USA	USD	-	Assessment of the cost of the disease	Direct costs	Patients with metastatic castration-sensitive prostate cancer
Borsoi, L. et al., 2023 [35]	Italy	EUR	-	Assessment of the cost of the disease	Direct costs	Patients with non-metastatic castration-resistant prostate cancer
Zhang, W. et al., 2023 [36]	Canada	USD	QALY	Assessment of the cost of the disease	Direct costs	Patients with prostate cancer
Lindsay, D. et al., 2024 [37]	Australia	AUD	-	Assessment of the cost of the disease	Direct costs	Patients with prostate cancer
Darba, J. et al., 2024 [38]	Spain	EUR	-	Assessment of the cost of the disease	Direct costs	Patients with prostate cancer
Kramer, K. et al., 2024 [39]	Germany	USD		Cost-effectiveness analysis	Direct costs	Patients with metastatic prostate cancer
Wu, D. et al., 2024 [40]	USA	USD	ICER, QALY	Systematic review	Direct costs	Patients with prostate cancer
Ax, A. et al., 2023 [41]	Sweden	EUR	QALY	Assessment of the cost of the disease	Direct costs	Patients with breast, colorectal, or prostate cancer
Edmunds, K. et al., 2025 [42]	Australia	AUD	SROI	Cost-effectiveness analysis	Direct costs	Patients with prostate cancer
Babela, R. et al., 2024 [43]	Slovakia	EUR	-	Assessment of the cost of the disease	Direct and indirect costs	Patients with prostate cancer
Alenizhad, F. et. Al., 2023 [44]	Iran	USD	-	Assessment of the cost of the disease	Direct and indirect costs	Patients with prostate cancer
Banafshe, D.T. et al., 2024 [45]	Iran	USD	-	Assessment of the cost of the disease	Direct and indirect costs	Patients with prostate cancer
Washington, S.L. et al., 2023 [46]	USA	Days off work	-	Assessment of the cost of the disease	Indirect costs	Patients with prostate cancer
Michel, K.F. et al., 2025 [47]	USA	USD	-	Assessment of the cost of the disease	Indirect costs	Patients with prostate cancer
Rice, H.E. et al., 2024 [48]	USA	Qualitative study	-	Assessment of the cost of the disease	Indirect costs	Patients with prostate cancer

INMB—Incremental Net Monetary Benefit; ICER—Incremental Cost-Effectiveness Ratio; QALY—Quality-Adjusted Life Year; SROI—Social Return on Investment.

**Table 4:** Summary of the results for treatments expressed as currency/QALY.

Treatment	Value
PARP inhibitors	CAD 565,383/QALY
Whole-genome-sequencing-guided systemic therapy	EUR 565,383/QALY
BRCA testing-guided olaparib	AUD 143,613/QALY
Androgen receptor pathway inhibitors	CAD 100,000/QALY
Rezvilutamide combined with androgen deprivation	USD 26,656.94/QALY
Enzalutamide	USD 6260/QALY

## Data Availability

All the data is presented in the article.
